# Prenatal Diagnosis Rate of Critical Congenital Heart Disease Remains Inadequate with Significant Racial/Ethnic and Socioeconomic Disparities and Technical Barriers

**DOI:** 10.1007/s00246-023-03262-2

**Published:** 2023-08-30

**Authors:** Arpine Davtyan, Heidi Ostler, Ian Fraser Golding, Heather Y. Sun

**Affiliations:** Division of Pediatric Cardiology, Rady Children’s Hospital and UC San Diego School of Medicine, 3020 Children’s Way, MC 5004, San Diego, CA 92123 USA

**Keywords:** Fetal cardiology, Prenatal diagnosis, Racial disparities, Congenital heart disease, Ultrasound

## Abstract

Prenatal diagnosis (preDx) of critical congenital heart disease (CCHD) decreases neonatal morbidity and mortality. Obstetrical fetal cardiac imaging guidelines in 2013 aimed to increase preDx. The objectives of this study were to determine the contemporary preDx rate of CCHD and identify maternal–fetal factors and variations in prenatal care that may be potential barriers*.* This retrospective single center study evaluated maternal demographics and characteristics of infants with CCHD (requiring cardiac catheterization or surgical intervention before 6 months-old) between 2016 and 2019. 58% of the 339 infants with CCHD had preDx. Infants with preDx were more likely to have mothers ≥ 35 years-old (*p* = 0.028), family history of CHD (*p* = 0.017), health insurance (*p* = 0.002), or anatomic scan with perinatology (*p* < 0.001). Hispanic infants were less likely to have preDx (45.6%, *p* = 0.005). PreDx rates were higher in infants with extracardiac/genetic anomalies (*p* < 0.001) and significantly different between CCHD subtypes (76% for single ventricle, 51% for biventricular/four-chamber view, 59% for proximal outflow tract anomalies, and 48% for distal great artery anomalies; *p* = 0.024). In infants without preDx, 25% of their mothers had indication for, but did not undergo, fetal echocardiography. PreDx rates of CCHD remains inadequate across subtypes detectable by standard fetal cardiac screening views, particularly in uninsured and Hispanic communities.

## Introduction

Congenital heart disease (CHD) is the most common congenital anomaly affecting approximately 1% of live births [1]. Critical congenital heart disease (CCHD) is a subset of CHD consisting of lesions that require early surgical or transcatheter intervention during infancy to allow for survival. Infants with critical congenital heart disease are at risk for increased rates of morbidity and mortality if not medically supported in a timely manner after birth [2]. Prenatal diagnosis of CCHD allows for multidisciplinary coordination of care, counseling, parental decision making, genetic testing, and psychosocial support. Risk stratification of prenatally diagnosed patients determines appropriate levels of care, with decisions about timing and mode of delivery, and if needed, allows immediate postnatal access to specialists, such as neonatologists, pediatric cardiologists, and pediatric cardiovascular surgeons [3]. Prenatally diagnosed fetuses with CCHD that are transferred for delivery at or near higher level pediatric cardiac centers have improved outcomes with decreased morbidity [4–7] and lower overall healthcare costs [8].

Prenatal diagnosis of CHD relies on detection by obstetric ultrasound and confirmation by fetal echocardiography. A fetal echocardiogram is recommended in pregnancies at risk for CHD based on maternal and/or fetal indications [9]. However, the majority of CHD occurs in low risk pregnancies, [10, 11] and thus prenatal diagnosis requires suspicion for fetal cardiac anomaly on screening obstetric ultrasounds. The detection of or suspicion for CHD requires careful evaluation of standard fetal cardiac views. Certain anomalies, such as hypoplastic left heart syndrome, are detectable in a standard four-chamber view. Other anomalies such as tetralogy of Fallot, transposition of the great arteries, and coarctation of the aorta may have normal appearing four-chamber views and require additional views involving the cardiac outflow tracts and/or great arteries. CCHD, in the vast majority of conditions, involves anomalies of the outflow tracts. In 2013, the American Institute of Ultrasound Medicine, American College of Obstetrics and Gynecology, American College of Radiology and Society of Radiologists in Ultrasound released new guidelines for routine obstetric anatomic ultrasounds to include views of the right and left cardiac outflow tracts in addition to the previously recommended four-chamber view of the fetal heart [12]. These updated guidelines were released based on previous studies showing low detection rates of CCHD in the United States with routine obstetrical ultrasounds, ranging from 12 to 53% [5]. Since the new guidelines were released, studies have shown no significant improvement in prenatal detection rates of critical outflow tract anomalies [13]. A prior retrospective analysis of patients seen at Rady Children’s Hospital compared pre- 2013-guideline and post-guideline detection rates of critical outflow tract anomalies, demonstrated that the prenatal detection rate increased only from 52 to 61%, and hypothesized that the low increase in detection rates was due to suboptimal obstetrical implementation and training on the updated guidelines [13]. If a mother is appropriately referred for a fetal echocardiogram, a detailed fetal echocardiogram performed by a specialized fetal cardiac sonographer or cardiologist and reviewed by a pediatric cardiologist with specialized training in advanced imaging results in highly accurate diagnosis of CHD [14, 15] and appropriate prenatal multidisciplinary care. This study seeks to determine the contemporary rate of prenatal diagnosis in infants with CCHD and identify maternal–fetal factors and variations in prenatal care that may be potential barriers to prenatal diagnosis.

## Methods

### Study Design/Subject Selection

This retrospective single center study evaluated maternal demographics and characteristics of infants with CCHD (defined as requiring cardiac catheterization or surgical intervention before 6 months-old) seen at Rady Children’s Hospital (RCHSD)/University of California, San Diego (UCSD) from October 2016 to December 2019. Eligible mother/infant pairs were identified through the RCHSD Heart Institute cardiac catheterization, cardiovascular surgery, and echocardiogram databases in addition to electronic medical record queries. Data collection was performed through retrospective chart review of the infant and mother’s medical records at RCHSD/UCSD. Prospective telephone surveys ascertained prenatal care information from mothers of neonates who underwent or died prior to intervention within the first 31 days of life. All cardiac defects were included with the exception of infants with isolated ventricular septal defect (VSD), vascular ring, or branch pulmonary artery anomalies, who were excluded. CCHD diagnoses were categorized according to subtypes and the fetal cardiac view required for detection and whether the infant had a single ventricle or two ventricle lesion (Table 1). Diagnoses such as total anomalous pulmonary venous connection and atrioventricular canal defects are considered biventricular/four-chamber view (4CV) lesions that require the four-chamber view for diagnosis. Diagnoses such as hypoplastic left heart syndrome and tricuspid atresia are categorized as single ventricle lesions that require the four-chamber view for diagnosis. Tetralogy of Fallot and d-transposition of the great arteries are examples of proximal outflow tract diagnoses requiring outflow tract views. Interrupted aortic arch and coarctation of the aorta are diagnoses categorized as distal outflow tract anomalies requiring the three vessel view. Maternal demographic information and prenatal care factors, such as maternal age at delivery, maternal BMI, highest level of prenatal care, estimated gestational age at start of prenatal care, whether or not an anatomy scan was performed in the second trimester, maternal medical problems, insurance status and type of insurance, annual household income during pregnancy and delivery location were collected from the medical record and phone survey. Area Deprivation Index (ADI), a validated measure of neighborhood socioeconomic disadvantage, was assigned according to maternal zip code [16]. The study was approved by the RCHSD/UCSD Institutional Review Board.

**Table 1:** Characteristics of congenital heart defects in study population, by subtype and fetal views required for diagnosis

CHD Subtype *(Fetal View Required for Diagnosis)*	Lesion	Number (% of Total Study Population)	Prenatally Diagnosed, n (%)	Postnatally Diagnosed, n (%)
Single Ventricle *(Four Chamber)*	Complex Single Ventricle Lesions	21 (6.2%)	17 (81%)	4 (19%)
	Hypoplastic left heart syndrome	17 (5%)	12 (70.6%)	5 (29.4%)
	Tricuspid atresia	4 (1.2%)	3 (75%)	1 (25%)
Biventricular / 4CV (*Four Chamber*)	Atrioventricular septal defect	19 (5.6%)	15 (79%)	4 (21%)
	Total anomalous pulmonary venous return	17 (5%)	2 (11.8%)	2 (88.2%)
	Ebstein anomaly	5 (1.5%)	4 (80%)	1 (20%)
Proximal Outflow Tract (*Outflow Tracts*)	Tetralogy of Fallot, pulmonary stenosis	42 (12.4%)	26 (62%)	16 (38%)
	Tetralogy of Fallot, pulmonary atresia	7 (2.1%)	5 (71.4%)	2 (28.6%)
	D-Transposition of the great arteries	41 (12%)	26 (63.4%)	15 (36.6%)
	Pulmonary stenosis	33 (9.7%)	12 (36.4%)	21 (63.6%)
	Double outlet right ventricle	27 (8%)	19 (70.4%)	8 (29.6%)
	Pulmonary atresia/intact ventricular septum	16 (4.7%)	10 (62.5%)	5 (37.5%)
	Aortic stenosis	8 (2.4%)	4 (50%)	4 (50%)
	Truncus arteriosus	8 (2.4%)	6 (75%)	2 (25%)
	Congenitally corrected transposition of the great arteries	2 (0.6%)	1 (50%)	1 (50%)
Distal Outflow Tract (*Three Vessel Trachea*)	Coarctation of the aorta	61 (18%)	29 (47.5%)	32 (52.5%)
	Interrupted aortic arch	9 (2.6%)	6 (66.7%)	3 (33.3%)
	Aortopulmonary window	2 (0.6%)	0 (0%)	2 (100%)

### Data Analysis

Continuous variable clinical demographic data was compared using the Wilcoxon Rank Sum Test. Categorical data was compared using Fisher’s exact tests. A *p*-value of less than 0.05 was used to define statistical significance. Univariable and multivariate regression analysis were performed to evaluate each predictor’s effect on the outcome of prenatal diagnosis. All statistical analyses were performed using R Core Team (2020. R: A language and environment for statistical computing. R Foundation for Statistical Computing, Vienna, Austria. (https://www.R-project.org/). Analyses were performed for the entire study cohort and for those requiring intervention in the first 31 days of life.

## Results

**Fig. 1:**
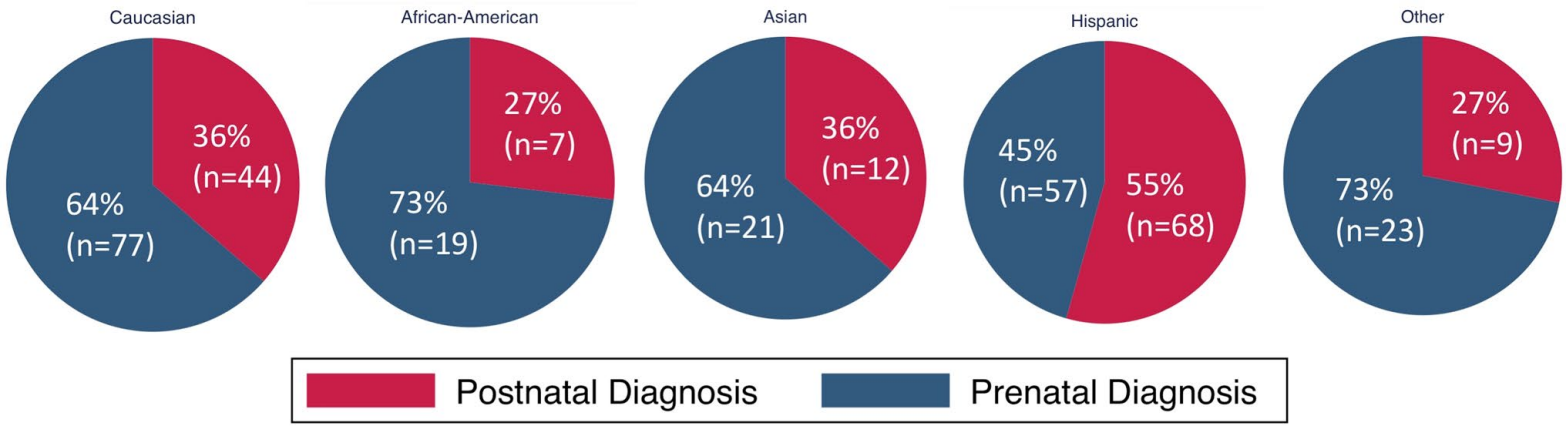
Prenatal diagnosis rates based on the infant’s race/ethnicity

**Fig. 2:**
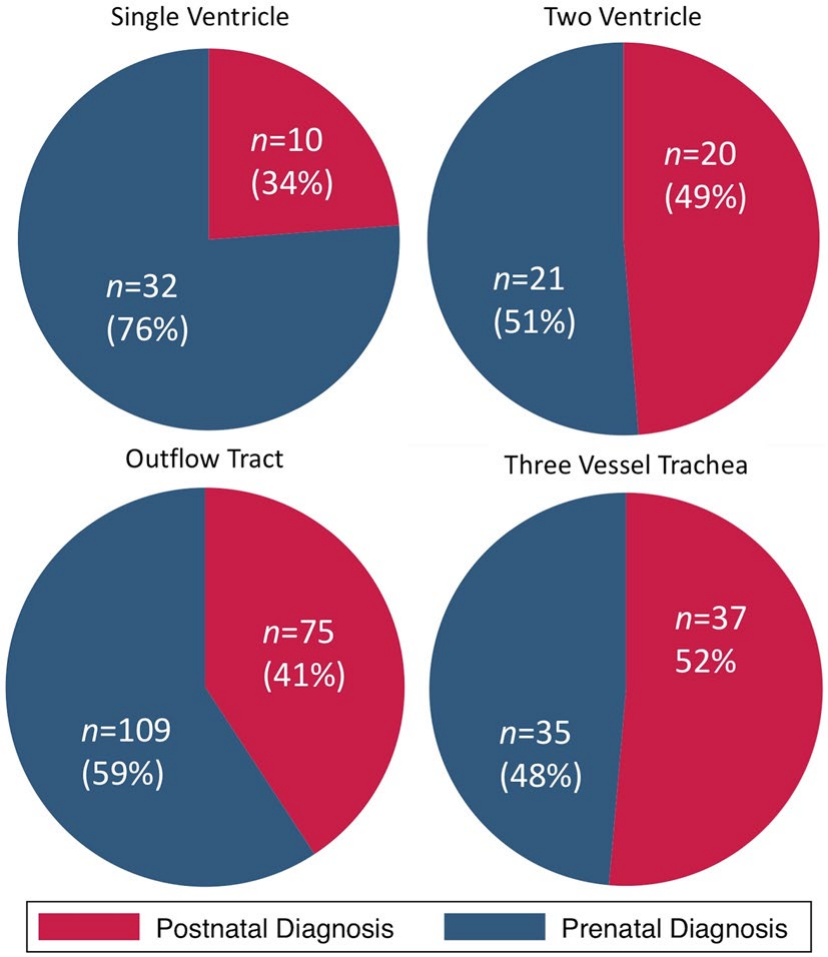
Prenatal diagnosis rates based on type of congenital heart disease. The single ventricle group includes diagnoses such as hypoplastic left heart syndrome that require the four-chamber view for diagnosis. The two ventricle group includes biventricular/4CV lesions that require the four-chamber view for diagnosis such as total anomalous pulmonary venous return. Tetralogy of Fallot and D-Transposition of the Great Arteries are examples of diagnoses requiring outflow tract views. Interrupted aortic arch and coarctation of the aorta were diagnoses categorized as requiring the three vessel trachea view

Overall, 58% (197/339) of infants with CCHD had prenatal diagnosis. The most common CCHD lesions were coarctation of the aorta (47% diagnosed prenatally), tetralogy of Fallot variants (63% diagnosed prenatally), and d-transposition of the great arteries (63% diagnosed prenatally) (Table 1). Infants with prenatal diagnosis were more likely to have mothers who were 35 or older (*p* = 0.028), had family history of CHD (*p* = 0.017), had health insurance (*p* = 0.002), or saw a perinatologist for their anatomic scan (*p* < 0.001) (Table 2). Hispanic infants were less likely to have prenatal diagnosis (57/125, 45.6%) compared to African American (19/26, 73%), Asian (21/33, 63.6%), or Caucasian/Non-Hispanic (77/121, 63.6%) infants (*p* = 0.005) (Fig. 1). Hispanic mothers were also more likely to have public health insurance (62%) compared to African American (48%), Asian (31%), or Caucasian/Non-Hispanic (18%) mothers (*p* < 0.001). In all infants who had prenatal diagnosis versus those who did not, there was no significant difference between maternal primary language, BMI, annual household income, socioeconomic status (based on ADI), health insurance type, or distance from a fetal cardiologist. Prenatal diagnosis rates were higher in infants with prenatal diagnosis of extracardiac/genetic anomalies (*p* < 0.001) and significantly different between subtypes of CCHD (76% in infants with single ventricle anomalies, 51% in infants with biventricular/4CV anomalies, 59% in infants with proximal outflow tract anomalies, and 48% in infants with anomalies of the distal great arteries; *p* = 0.024) (Table 3, Fig. 2).

**Table 2:** Rates of prenatal diagnosis by maternal/fetal factors

	All study subjects			Subgroup (age < 31 days at intervention)		
	Prenatal diagnosis			Prenatal diagnosis		
	No	Yes	*p*-value	No	Yes	*p*-value
Primary language						
English	122 (41.8%)	170 (58.2%)	1.000	76 (40.4%)	112 (59.6%)	0.150
Other	20 (42.6%)	27 (57.4%)		15 (55.6%)	12 (44.4%)	
Maternal age at delivery						
< 35 Years	115 (44.9%)	141 (55.1%)	**0.028**	76 (45.0%)	93 (55.0%)	0.086
≥ 35 Years	25 (30.9%)	56 (69.1%)		13 (29.5%)	31 (70.5%)	
Maternal BMI	25.4 (21.8 -29.8)	24.4 (21.6–30)	0.737	26.4 (22.5–31)	24.5 (21.6–30.7)	0.311
Prenatal Care						
No	5 (100%)	0(0%)	**0.011**	5 (100%)	0 (0%)	**0.012**
Yes	133 (40.3%)	197 (59.7%)		85 (40.7%)	124 (59.3%)	
Start of prenatal care (EGA)	8 (6–12)	9 (7–12)	0.600	8 (6–12)	8 (7–12)	0.833
Highest level of prenatal care						
Midwife	6 (85.7%)	1 (14.3%)	** < 0.001**	6 (85.7%)	1 (14.3%)	** < 0.001**
Family Medicine	4 (100%)	0 (0%)		4 (100%)	0 (0%)	
Obstetrician	72 (65.5%)	38 (34.5%)		52 (71.2%)	21 (28.8%)	
Perinatologist	18 (10.7%)	151 (89.3%)		8 (7.3%)	101 (92.7%)	
Anatomy Scan Done In 2nd Trimester?						
No	5 (83.3%)	1 (16.7%)	**0.021**	5 (83.3%)	1 (16.7%)	**0.028**
Yes	93 (33.9%)	181 (66.1%)		64 (35.8%)	115 (64.2%)	
Maternal Medical Problem						
No	88 (44.4%)	110 (55.6%)	0.263	53 (43%)	70 (57%)	0.780
Yes	53 (37.9%)	87 (62.7%)		37 (40.7%)	54 (59.3%)	
Extracardiac/Genetic Abnormality Diagnosed Prenatally						
No	110 (47%)	124 (53%)	** < 0.001**	73 (45.3%)	88 (54.7%)	**0.007**
Yes	19(21.1%)	71 (78.9%)		11 (23.4%)	36 (76.6%)	
Fetal Echocardiogram Done?						
No	126 (99.2%)	1(0.8%)	** < 0.001**	83 (97.6%)	1 (0.8%)	** < 0.001**
Yes	5(2.5%)	194(97.5%)		2 (1.6%)	123 (98.4%)	
Miles from fetal cardiologist	20(10.00–35.00)	16(9.00–28.00)	0.102	21 (10.50–34.00)	16.50 (9.00–28.00)	0.137
Interpreter of Fetal Echo, If Done						
Perinatology + Fetal Cardiology	0 (0%)	20 (100%)	** < 0.001**	0 (0%)	11 (100%)	**0.026**
Fetal Cardiology	1 (0.060%)	169 (99.4%)		1 (0.9%)	110 (99.1%)	
Perinatology	3 (60.0%)	2(40.0%)		0 (0%)	2 (100%)	
Not Sure/Other	1 (33.3%)	2(66.7%)		1 (100%)	0 (0%)	
Family History Of CHD						
No	133 (43.3%)	174 (56.7%)	**0.017**	86 (44.3%)	108 (55.7%)	**0.009**
Yes	3 (15%)	17 (85%)		1 (7.1%)	13 (92.9%)	
Insurance During Pregnancy						
No	9 (90.0%)	1(1.0%)	**0.002**	6 (100%)	0 (0%)	**0.005**
Yes	132 (40.2%)	196 (59.8%)		85 (40.7%)	124 (59.3%)	
Insurance Type						
Medi-cal	47 (42.0%)	65 (58.0%)	0.260	35 (47.9%)	38 (52.1%)	**0.035**
Military	19 (52.8%)	17 (47.2%)		16 (57.1%)	12 (42.9%)	
Other state public	7 (29.2%)	17 (70.8%)		4 (26.7%)	11 (73.3%)	
Private	59 (37.8%)	97 (62.2%)		30 (32.3%)	63 (67.7%)	
Annual Household Income During Pregnancy						
0–49,000	50 (39.4%)	77(60.6%)	0.562	38 (42.2%)	50 (56.8%)	0.471
50,000–79,000	5 (41.7%)	7 (58.3%)		5 (41.7%)	7 (58.3%)	
80,000–99,000	7 (46.7%)	8(53.3%)		7 (46.7%)	8 (53.3%)	
100,000 +	5 (25.0%)	15(75.0%)		5 (25.0%)	15 (75.0%)	
ADI	18 (11.0 -28.00)	16 (9.00–26.00)	0.105	20 (12.00–29.00)	16.00 (9.00–26.00)	**0.028**
Infant’s Race/Ethnicity						
African–American	7 (26.9%)	19 (73.1%)	**0.005**	5(29.4%)	12 (70.6%)	**0.008**
Asian	12 (36.4%)	21 (63.6%)		6 (31.6%)	13 (68.4%)	
Caucasian, Non- Hispanic	44 (36.4%)	77 (63.6%)		27 (35.1%)	50 (64.9)	
Hispanic	68 (54.4%)	57 (45.6%)		48 (57.8%)	35 (42.2%)	
Other	9 (28.1%)	23 (71.9%)		5 (26.3%)	14 (73.7%)	
Delivery Location						
Hospital	138 (100%)	196 (99%)	0.072	88 (41.5%)	124 (58.5%)	0.176
Home	1(100%)	0 (0%)		0 (0%)	0 (0%)	
Birthing Center	2(1.0%)	0 (0%)		2 (100%)	0 (0%)	
Mortality pre-cardiac intervention						
Yes	2 (1.4%)	3 (1.5%)	1	0	0	–
No	140 (98.6%)	194 (98.5%)		91 (100%)	124 (100%)	
Mortality post-cardiac intervention						
Yes	7 (5%)	11 (5.6%)	1	7 (7.7%)	7 (5.6%)	0.585
No	134 (95%)	194 (98.5%)		84 (92.3%)	117(94.4%)	

Bold values are statistically significant (*p* < 0.05)

**Table 3:** Rates of prenatal diagnosis by lesion and echocardiographic view required for diagnosis

	Prenatal diagnosis					
	All study subjects			Subgroup (Age < 31 days at INtervention)		
	No	Yes	*p*-value	No	Yes	*p*-value
Four Chamber View (single ventricle CHD)	10 (23.8%)	32 (76.2%)	**0.024**	9 (29%)	22 (71%)	**0.009**
Four Chamber View (biventricular CHD)	20 (48.8%)	21 (51.2%)		14 (77.8%)	4 (22.2%)	
Outflow Tract View	75 (40.8%)	109 (59.2%)		43 (40.2%)	64 (59.8%)	
Three Vessel Trachea View	37 (51.4%)	35 (48.6%)		25 (42.4%)	34 (57.6%)	

Bold values are statistically significant (*p* < 0.05)

**Fig. 3:**
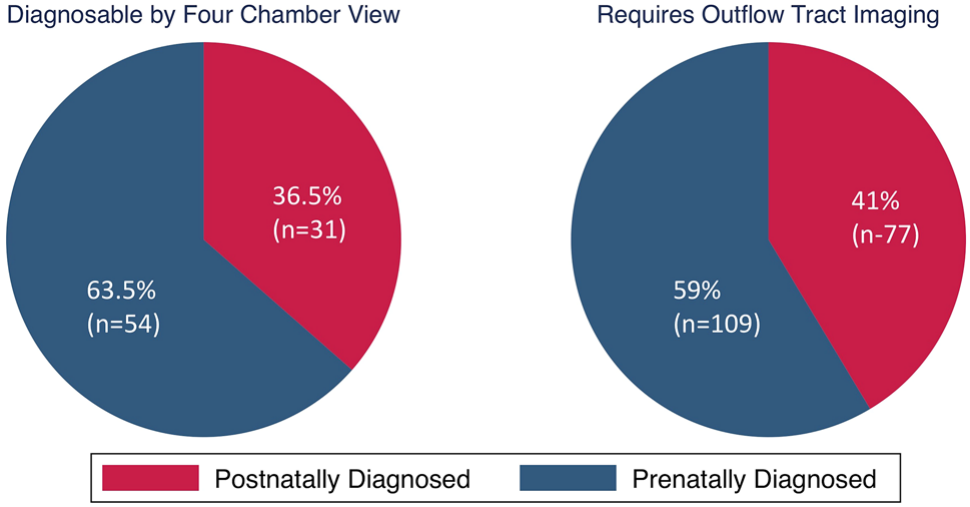
Prenatal diagnosis rates based on fetal cardiac view required for diagnosis

In the infants without prenatal diagnosis, 73% should have been detected on screening cardiac views on anatomy ultrasound: 31/142 (21%) had CCHD detectable by fetal four-chamber view and 77/142 (54%) had CCHD detectable by adequate outflow tract imaging (Fig. 3). 25% (36/142) of mothers of infants without prenatal diagnosis had indication for, but were not referred for or did not undergo, fetal echocardiography; of these mothers, 47% (17/36) had diabetes (at least 6 with pre-gestational diabetes; 11 mothers had insufficient recall/data to determine type/severity of diabetes), 14% (5/36) were carrying monochorionic twin pregnancies, and 36% (13/36) had a fetus with an extracardiac (5 renal, 2 cleft lip/palate, 1 brain, 1 gastrointestinal) or genetic (Trisomy 21) anomaly, 2/36 had incomplete screening fetal cardiac views, and 1 had an IVF pregnancy.

Of the neonatal subgroup that required/underwent intervention within the first 31 days of life, 58% (124/215) were prenatally diagnosed. Within this subgroup, the statistical analysis yielded the same statistically significant factors as those in the whole study group with three exceptions. Mothers with public health insurance (*p* = 0.03) or a lower socioeconomic status as determined by the ADI (*p* = 0.028) were less likely to have an infant with prenatal diagnosis. Mother’s age at delivery did not reach statistical significance. Within the neonatal subgroup, infants of mothers who had prenatal care (*p* = 0.012), had insurance (*p* = 0.005), saw a perinatologist as the highest level of care (*p* < 0.001), had an anatomy scan done in the second trimester (*p* = 0.028), had a fetal echocardiogram (*p* < 0.001) were also more likely to be diagnosed prenatally.

In all infants who had prenatal diagnosis versus those who did not, there was no significant difference between maternal primary language, gestational age at start of prenatal care, presence of maternal medical problems, socioeconomic status, or miles from a fetal cardiologist.

In the univariate analysis (Table 4), Hispanic infants were less likely to be diagnosed prenatally (OR 0.48, *p* = 0.005 for the entire study population, OR 0.39, p = 0.004 for the neonatal subgroup). Mothers older than 35 years were more likely to have an infant with prenatal diagnosis (OR 1.83, *p* = 0.028). Although there was no statistically significant difference between insurance types when analyzing the entire study population, those with private insurance were more likely to be prenatally diagnosed in the neonatal intervention subgroup (OR 1.9, *p* = 0.04). Mothers with an obstetrician, midwife or family practice practitioner as their highest level of prenatal care were less likely to have an infant with prenatal diagnosis (OR 0.06, *p* < 0.001 for the entire study population; OR 0.03, *p* < 0.001 for the neonatal subgroup) compared to those who had a perinatologist as the highest level of care. Those with their anatomic scan at a perinatology office were more likely to be prenatally diagnosed as compared to those with their scan at an obstetric office (OR 3.35, *p* < 0.001 for the entire study population; OR 3.2, *p* = 0.003 for the neonatal subgroup).

**Table 4:** Univariable logistic regression

Variable	All study subjects				Subgroup (Age < 31 days at intervention)		
	OR	P	CI		OR	P	CI
View required for diagnosis and single ventricle vs biventricular							
Four chamber view (biventricular CHD), reference	1				1		
Four chamber view (single ventricle HCD)	3.04	**0.02**	1.20–8.03		8.55	**0.002**	2.37–37.23
Outflow tract view	1.38	0.35	0.69–2.74		5.21	**0.006**	1.74–19.35
Three vessel trachea view	0.90	0.79	0.42–1.94		4.76	**0.013**	1.50–18.39
Primary Language							
English, reference	1				1		
Other	0.97	0.92		0.52–1.83	0.54	0.14	0.24–1.22
Mother’s age at delivery							
Age < 35 years, reference	1				1		
Age > 35 years	1.83	**0.028**		1.08–3.15	1.95	0.07	0.97–4.10
Maternal BMI	0.99	0.73		0.96–1.03	0.98	0.48	0.94–1.03
Start of prenatal care (EGA)	0.98	0.30		0.93–1.02	0.95	0.09	0.91–1.01
Maternal Medical Problem							
No, reference	1				1		
Yes	1.31	0.23		0.85–2.05	1.11	0.72	0.64–1.92
Extracardiac/Genetic Abnormality Diagnosed Prenatally							
No	1				1		
Yes	3.31	** < 0.001**		1.91–5.98	2.72	**0.008**	1.33–5.40
Location of anatomic scan							
Obstetrician/other, reference	1				1		
Perinatology office	3.35	** < 0.001**		1.78–6.39	3.21	**0.003**	1.50–7.05
Radiology office	1.21	**0.622**		0.57–2.59	1.14	0.76	0.49–2.66
ADI	0.99	0.16		0.98–1.00	0.99	0.08	0.97–1.00
Miles from fetal cardiologist	0.98	0.32		0.99–1.00	1	0.39	0.99–1.00
Highest level of prenatal care							
Perinatologist, reference	1				1		
Obstetrician, midwife, family practice	0.06	** < 0.001**		0.03–0.10	0.03	** < 0.001**	0.01–0.06
Infant’s race/ethnicity							
Caucasian/white, non-Hispanic, reference	1				1		
African-American	1.55	0.36		0.63–4.23	1.30	0.66	0.43–4.42
Asian	1	1		0.45–2.28	1.17	0.77	0.41–3.65
Hispanic	0.48	**0.005**		0.29–0.80	0.39	**0.004**	0.21–0.74
Other	1.46	0.38		0.64–3.59	1.51	0.47	0.52–5.08
Insurance type							
Medi-cal, reference	1				1		
Military	0.65	0.26		0.30–1.38	0.69	0.41	0.29–1.66
Other state public	1.76	0.25		0.70–4.85	2.53	0.14	0.78–9.81
Private	1.19	0.49		0.72–1.95	1.9	**0.04**	1.03–3.66

Bold values are statistically significant (*p* < 0.05)

Infants with single ventricle CHD were more likely to be prenatally diagnosed compared with biventricular/4CV CHD (OR 3.05, *p* = 0.02). In the neonatal intervention subgroup, those with single ventricle lesions (OR 8.55, *p* = 0.002), proximal outflow tract abnormalities (OR 5.2, *p* = 0.006), and distal outflow tract abnormalities (OR 4.76, *p* = 0.013) were more likely to be prenatally diagnosed as compared to biventricular/4CV lesions. Proximal outflow tract abnormalities included pulmonary stenosis, aortic stenosis, d-transposition of the great arteries, truncus arteriosus and tetralogy of Fallot. Distal outflow tract abnormalities included coarctation of the aorta and interrupted aortic arch. Those with extracardiac/genetic anomalies were more likely to be prenatally diagnosed for both the entire study population (OR 3.3, *p* < 0.001) and the neonatal subgroup (OR 2.7, *p* = 0.008).

The multivariate analysis (Table 5) included infant race/ethnicity, highest level of maternal prenatal care, location of anatomic scan, extracardiac/genetic abnormalities, views required for diagnosis, and single ventricular lesions versus biventricular/4CV lesions. Several variables were excluded from the multivariate analysis due to small subgroups. The multivariate analysis confirmed that Hispanic infants were less likely to be prenatally diagnosed (OR 0.42, *p* = 0.048). Those who had an obstetrician, midwife or family practice physician as their highest level of prenatal care were less likely to be prenatally diagnosed (OR 0.08, *p* < 0.001) compared to those who saw a perinatologist. Those who had their anatomic scan at a perinatology office were more likely to be prenatally diagnosed (OR 2.89, *p* = 0.01). The subtype of CCHD (single ventricle vs biventricular/4CV CCHD vs outflow tract anomalies vs distal great artery anomalies) and present of fetal extracardiac/genetic abnormality diagnosed were not statistically significant in the multivariate analysis.

**Table 5:** Multivariable regression analysis

Variable	All study subjects			
	OR	P	CI	
View required for diagnosis and single ventricle vs biventricular				
Four chamber view (biventricular CHD), reference	1			
Four chamber view (single ventricle CHD)	2.99	0.16	0.68–14.31	
Proximal outflow tract view	1.32	0.64	0.41–4.13	
Three vessel trachea view	1.11	0.87	0.30–4.13	
Extracardiac/Genetic Abnormality Diagnosed Prenatally				
No	1			
Yes	2.10	0.11		0.86–5.51
Location of anatomic scan				
Obstetrician/other, reference	1			
Perinatology office	2.89	**0.01**		1.26–6.81
Radiology office	1.89	0.22		0.69–5.31
Highest level of prenatal care				
Perinatologist, reference	1			
Obstetrician, midwife, family practice	0.08	** < 0.001**		0.03–0.16
Infant’s race/ethnicity				
Caucasian/white, non-Hispanic, reference	1			
African–American	0.48	0.35		0.11–4.50
Asian	1.28	0.71		0.35–5.21
Hispanic	0.42	**0.048**		0.18–0.98
Other	2.58	0.28		0.61–14.16

Bold values are statistically significant (*p* < 0.05)

## Discussion

Overall, only 58% of infants with CCHD were diagnosed prenatally. This study found important demographic factors associated with a lower likelihood of prenatal diagnosis. Hispanic patients, those with lower socioeconomic status, and those without insurance were less likely to have prenatal diagnosis. Hispanic infants in our study were less likely to be prenatally diagnosed, with an odds ratio of 0.42 in a multivariate analysis after accounting for highest level of obstetrical care, location of anatomic scan, presence of extracardiac/genetic anomalies, and subtype of CHD. Although there was no statistically significant difference in level of prenatal care and lack of health insurance in Hispanic mothers, there may be other factors that are more difficult to define such as cultural differences that are not accounted for in our analysis. Infants who required neonatal intervention were less likely to be prenatally diagnosed if they had lower socioeconomic status. Socioeconomic status as measured by ADI was not statistically significant when analyzing the entire study population. We also found no statistically significant difference in household income between the prenatal versus postnatal diagnosis groups, but our dataset was limited by the retrospective nature of this study.

The findings in our study are consistent with multiple single and multicenter studies in the US and Canada linking lower socioeconomic status with decreased rates of prenatal diagnosis. A large multicenter Fetal Heart Society collaborative which demonstrated that Hispanic ethnicity, lower socioeconomic status, and rural residence were associated with decreased prenatal diagnosis in infants with TGA and lower socioeconomic status was associated with decreased prenatal diagnosis in infants with HLHS in the United States and Canada [17]. Another single center study in Wisconsin found that those living in impoverished or rural communities had lower rates of prenatal diagnosis.[18] A single center study in the United States found that patients with public health insurance and lower socioeconomic status were less likely to have prenatal diagnosis as compared to patients with private insurance and higher socioeconomic status [19]. A recent study from Alberta, Canada found that distance from a tertiary care center and lower socioeconomic status were associated with lower likelihood of prenatal diagnosis [20]. Even in places with universal health insurance, such as Canada, those of lower socioeconomic status were less likely to be prenatally diagnosed, suggesting that lower socioeconomic status has a role to play that is independent of insurance status. In our study, mothers without health insurance were less likely to have infants with prenatal diagnosis. Infants with CCHD who required neonatal intervention in our study were more likely to be prenatally diagnosed if they had private health insurance. However, we found no statistically significant different in type of insurance between those with or without prenatally diagnosis when analyzing the entire study population. A single center study at Northwestern University found that women with public health insurance were less likely to undergo diagnosis testing after positive screening for aneuploidy [21], suggesting that mothers without health insurance or with public health insurance may have additional challenges with understanding and navigating a complex multidisciplinary prenatal care system. Mothers without health insurance or with public health insurance may have limited resources to access advanced medical evaluation with a perinatologist and/or fetal echocardiography.

The overall 58% prenatal diagnosis rate of CCHD in San Diego County remains inadequate despite the 2013 societal guidelines intended to increase prenatal detection rates of CHD, with inclusion of views of the cardiac outflow tracts in addition to the four-chamber view of the fetal heart during routine obstetric anatomic ultrasounds [12]. The four-chamber view is the most standard and familiar fetal cardiac view to obstetric providers, and yet prenatal diagnosis rate of CCHD diagnosable by even this view remained a suboptimal 64% in this contemporary study. In CCHD requiring outflow tract imaging for diagnosis, the prenatal diagnosis rate was even lower at 59% in this study.

Worldwide prenatal diagnosis rates of CCHD are variable, with some regions in the world having prenatal diagnosis rates as high as 87% [22, 23]. Prior to 2013, a study analyzing the Society of Thoracic Surgeons database between 2006 and 2012 across 91 U.S. centers found a prenatal diagnosis rate of 34% for all CHD requiring surgical intervention at age less than or equal to six months; the detection rate was highest at 67% for hypoplastic left heart syndrome and lowest at 9% for total anomalous pulmonary venous return [5]. Similarly, a single center study in the United States found lower rates of prenatal diagnosis in CHD that required additional views other than four-chamber view, with a prenatal diagnosis rate of 48% for CHD diagnosable by 4 chamber view and 36% for CHD requiring outflow tract views [18]. A regional study in the Netherlands from 2002 to 2012 noted significantly higher prenatal detection rates (93%) in single ventricle lesions, with an overall CHD prenatal detection rate of 59% [24]. In Alberta, Canada between 2008 and 2018, 58% of major CHD was diagnosed prenatally with higher detection rates of 82% for lesions requiring the four-chamber view versus those 66% of those requiring the outflow tract view [25]. More recent studies done after the 2013 modified guidelines [12] in the United States and internationally have continued to show variable and wide ranges of prenatal diagnosis rates, with CHD diagnosable by the standard four-chamber view more often diagnosed prenatally (Table 6) [5, 22, 26, 27]. In San Diego between 2015 and 2016, prenatal detection rates of critical outflow tract anomalies was 61% [13]. In Japan between 2013 and 2017, 41% of infants with CCHD were diagnosed prenatally [28]. In Sweden between 2013 and 2017, 56% of infants with CCHD were diagnosed prenatally, with the prenatal detection rate for single ventricle lesions being 100% compared to 9% for TGA, an outflow tract anomaly [23]. In Copenhagen between 2015 and 2018, 89% of infants with CCHD were identified prenatally (although the study excluded those with pulmonary or aortic stenosis and total anomalous pulmonary venous return); 100% of single ventricle and HLHS infants had prenatal diagnosis compared to 82% of TGA infants [29].

**Table 6:** Prenatal diagnoses rates reported in different studies around the world

Author (Location)	Years of study	Overall prenatal diagnosis rate	Four chamber view	Outflow tract view	Single ventricle lesions	D-TGA	HLHS
Quartermain (USA)[5]	2006–2012	34%	57%	32%	n/a	n/a	67%
Campbell (12 States in USA)[26]	2007–2012	27.9%	n/a	n/a	n/a	n/a	n/a
Hill (Wisconsin, USA)[18]	2007–2013	61%	84%	56%	n/a	n/a	n/a
Kaur (Alberta, Canada) (25)	2008–2018	58%	75–88%	56–79%	n/a	n/a	n/a
Sun (San Diego, CA, USA)[13]	2010–2013	52%	n/a	52%	n/a	26%	n/a
Evans (Nevada, USA)[31]	2012–2021	66%	n/a	n/a	n/a	n/a	n/a
Matsui (Japan)[28]	2013–2017	41%	n/a	n/a	n/a	n/a	n/a
Waern (Sweden)[23]	2014–2016	56%	n/a	n/a	100%	9%	n/a
Sun (San Diego, CA, USA)[13]	2015–2016	61%	n/a	61%	n/a	24%	n/a
Vedel (Copenhagen, Denmark)[29]	2015–2018 ara>	89%	n/a	n/a	100%	82%	100%

*D-TGA* D-transposition of the great arteries, *HLHS* hypoplastic left heart syndrome

In our study, a significant percentage (25%) of mothers of infants without prenatal diagnosis did not undergo fetal echocardiography although they had an indication for one. Common missed indications were maternal diabetes, fetal extracardiac defects or genetic anomaly, or a monochorionic twin pregnancy. Our study noted certain factors associated with increased likelihood of prenatal diagnosis, such as family history of congenital heart disease and presence of an extracardiac/genetic fetal anomaly, that are recommended indications for referral for a fetal echocardiogram [9]. We propose that increasing awareness in obstetrical providers of these maternal–fetal indications for referral for fetal echocardiography would increase rates of prenatal diagnosis. If a mother is appropriately referred for and undergoes fetal echocardiography, there is a very high likelihood of an accurate prenatal CHD diagnosis, as a comprehensive fetal echocardiogram has been previously shown to result in highly accurate diagnosis of CHD [14, 15]. Our study demonstrated a strong association between having a perinatologist as the highest level obstetrical provider and prenatal diagnosis of CHD. Several factors noted to be associated with a higher likelihood of prenatal diagnosis, such as advanced maternal age and extracardiac anomalies, would also be indications for referral to a perinatologist. Evaluation of fetal cardiac anatomy by perinatology is likely to be more comprehensive, with increased sonographer and physician expertise and comfort in detecting abnormality.

### Limitations

This study is a single center study and findings may not be generalizable to larger populations. There is a significantly larger Hispanic population and smaller white, non-Hispanic population in San Diego County and our study population when compared to the United States as a whole [30]. Study data was obtained via retrospective chart review and cannot assess causality. Some data was obtained via telephone surveys of mothers of infants with CCHD and that data may be inaccurate due to recall bias, memory failure or other reasons. Telephone surveys for the entire study population were not obtained due lack of personnel time and resources; the survey was therefore limited to focus on those patients who required early intervention.

Due to the multiple referring obstetrical/perinatology practices outside of our institution’s accessible medical records and retrospective nature of the study, the study authors had limited data to certain aspect of prenatal care. This study did not include the total number of pregnancies diagnosed with CHD that terminated, as the information was not accurately attainable, and thus the true prenatal detection rate may be higher. Actual ultrasound images from the obstetric anatomic ultrasound scan were not available or acquired to review to determine whether the correct cardiac screening views were obtained and whether they were abnormal or normal. Reviewing obstetric ultrasound scan images in future studies may be helpful in addressing limitations in image technique and/or interpretation by providers obtaining anatomic ultrasound scans. Indications for referral to perinatology for anatomic scan (potentially resulting in increased detection of CHD) was not available. Despite these limitations, this study identified certain important factors strongly associated with lower likelihood of prenatal diagnosis that should be addressed in future outreach and policy making.

## Conclusion

Prenatal diagnosis rates of CCHD remains inadequate, particularly across subtypes detectable by the standard fetal cardiac screening views. Certain maternal factors, such as lack of health insurance, suboptimal prenatal care, and Hispanic ethnicity, are associated with a lower likelihood of prenatal diagnosis and addressing these factors should be prioritized. Outreach to primary obstetrical providers and education regarding fetal cardiac imaging and indications for fetal echocardiogram, particularly in uninsured and Hispanic communities, should be prioritized to increase rates of prenatal diagnosis of CCHD. Additional multidisciplinary investments should aim to bring fetal echocardiography services to lower socioeconomic communities and reduce the gap in accessing higher levels of advanced prenatal care.
